# *Histoplasma capsulatum* Isolated from *Tadarida brasiliensis* Bats Captured in Mexico Form a Sister Group to North American Class 2 Clade

**DOI:** 10.3390/jof7070529

**Published:** 2021-06-30

**Authors:** Tania Vite-Garín, Daniel A. Estrada-Bárcenas, David S. Gernandt, María del Rocío Reyes-Montes, Jorge H. Sahaza, Cristina E. Canteros, José A. Ramírez, Gabriela Rodríguez-Arellanes, Lisandra Serra-Damasceno, Rosely M. Zancopé-Oliveira, John W. Taylor, Maria Lucia Taylor

**Affiliations:** 1 Unidad de Micología, Departamento de Microbiología y Parasitología, Facultad de Medicina, Universidad Nacional Autónoma de México (UNAM), Ciudad de México 04510, Mexico; tania.vite.garin@gmail.com (T.V.-G.); remoa@unam.mx (M.d.R.R.-M.); jhsahaza@hotmail.com (J.H.S.); jarb@unam.mx (J.A.R.); batgaby@unam.mx (G.R.-A.); 2 Colección Nacional de Cepas Microbianas y Cultivos Celulares, Centro de Investigación y de Estudios Avanzados, Instituto Politécnico Nacional (CINVESTAV, IPN), Ciudad de México 07360, Mexico; destrada@cinvestav.mx; 3 Departamento de Botánica, Instituto de Biología, Universidad Nacional Autónoma de México (UNAM), Ciudad de México 04510, Mexico; dsgernandt@ib.unam.mx; 4 Departamento de Micología, Instituto Nacional de Enfermedades Infecciosas (INEI), Administración Nacional de Laboratorios e Institutos de Salud (ANLIS) “Dr. Carlos G. Malbrán”, Buenos Aires 1281, Argentina; cecanteros@gmail.com; 5 Centro de Ciências da Saúde, Departamento de Saúde Comunitária, Universidade Federal do Ceará, Fortaleza 60455-610, Brazil; lisainfecto@gmail.com; 6 Laboratório de Micologia, Setor Imunodiagnóstico, Instituto Nacional de Infectología Evandro Chagas, Fundação Oswaldo Cruz (FIOCRUZ), Rio de Janeiro 21040-360, Brazil; rosely.zancope@ini.fiocruz.br; 7 Department of Plant and Microbial Biology, University of California, Berkeley, CA 94720, USA; jtaylor@berkeley.edu

**Keywords:** *Histoplasma capsulatum*, bat host, NAm 3 clade, new lineage, phylogenetic reconstruction, concatenated sequence-types network

## Abstract

*Histoplasma capsulatum* is a dimorphic fungus associated with respiratory and systemic infections in mammalian hosts that have inhaled infective mycelial propagules. A phylogenetic reconstruction of this pathogen, using partial sequences of *arf*, *H-anti*, *ole1*, and *tub1* protein-coding genes, proposed that *H. capsulatum* has at least 11 phylogenetic species, highlighting a clade (BAC1) comprising three *H. capsulatum* isolates from infected bats captured in Mexico. Here, relationships for each individual locus and the concatenated coding regions of these genes were inferred using parsimony, maximum likelihood, and Bayesian inference methods. Coalescent-based analyses, a concatenated sequence-types (CSTs) network, and nucleotide diversities were also evaluated. The results suggest that six *H. capsulatum* isolates from the migratory bat *Tadarida brasiliensis* together with one isolate from a *Mormoops megalophylla* bat support a NAm 3 clade, replacing the formerly reported BAC1 clade. In addition, three *H. capsulatum* isolates from *T. brasiliensis* were classified as lineages. The concatenated sequence analyses and the CSTs network validate these findings, suggesting that NAm 3 is related to the North American class 2 clade and that both clades could share a recent common ancestor. Our results provide original information on the geographic distribution, genetic diversity, and host specificity of *H. capsulatum*.

## 1. Introduction

*Histoplasma capsulatum* is a pathogenic ascomycete that infects humans and other mammals. This fungus is distributed worldwide and is usually found in bird and bat droppings. Its saprobe and infective mycelial morphotype grow in environmental conditions that favor the production of aerosolized mycelial propagules, mainly microconidia and hyphal fragments that are inhaled by susceptible hosts, initiating respiratory and systemic infections.

Bats are able to develop natural and experimental histoplasmosis infections [1,2,3,4,5,6]. Infected bats could act as reservoirs and dispersers of *H. capsulatum* in favorable environments, playing a possible role in the incorporation of the fungus in new ecological niches [7,8,9].

Over the last three decades, *H. capsulatum* has been the subject of several genotyping studies that have engaged its DNA polymorphism using molecular tools such as restriction fragment length polymorphism and random amplified polymorphic DNA methods [10,11,12,13,14,15,16], analyses of individual and concatenated genes [17,18,19,20,21,22,23,24,25,26] and whole genomes [27,28], which have contributed to the knowledge of the genetic diversity and phylogeny of this pathogen. Currently, *H. capsulatum* consists of various groups of isolates that differ genetically and correlate with particular geographic distributions, which are considered as a complex of cryptic species [20,26]. Phylogenetic analyses using molecular markers have been a useful tool for species recognition and for studying the evolutionary genetics of microbial pathogens in the fields of the host−parasite relationship, epidemiology, and medicine [29,30].

In general, fungal species recognition is based on biological or morphological species concepts; however, the description of several cryptic species among micromycetes have been proposed by exploring different methods and concepts to delimit species, such as the phylogenetic species concept and its derivatives [29]. In regard to the *H. capsulatum* species delimitation, the genealogical concordance phylogenetic species recognition (GCPSR) concept, mentioned by Taylor et al. [29], is one of the most validated concepts because it allows the analysis of micromycetes with certain characteristics, highlighting the lack of morphological characters, the absence of sexual spores, and the heterothallic species.

Kasuga et al. [20] studied the phylogenetic relationships of 137 *H. capsulatum* isolates from 25 countries and interpreted the results by applying the GCPSR concept. They used multilocus sequence typing (MLST) analyses of partial DNA sequences of four protein-coding genes: ADP ribosylation factor (*arf*), H-antigen precursor (*H-anti*), delta-9 fatty acid desaturase (*ole1*), and alpha-tubulin (*tub1*). Based on the analyses of these isolates with different geographical origins and sources, they identified eight *H. capsulatum* clades corresponding to genetically distinct geographical populations: North American class 1 (NAm 1), North American class 2 (NAm 2), Latin American group A (LAm A), Latin American group B (LAm B), Australian, Netherlands, Eurasian, and African. Seven of these clades (NAm 1, NAm 2, LAm A, LAm B, Australian, Netherlands, and African) were recognized as phylogenetic species belonging to the *H. capsulatum* complex. These authors also proposed the existence of seven lone lineages, which delimit an isolate or a small group of isolates that have a single multilocus genotype. Taylor et al. [21], using the same molecular markers, suggested the existence of a particular clade of *H. capsulatum* isolates, which contained one isolate from a *Mormoops megalophylla* bat (Chiroptera: Mormoopidae) and two isolates recovered from different tissues of the free-tailed bat, *Tadarida brasiliensis* (Chiroptera: Molossidae), all captured in Mexico. Later, Vite-Garín et al. [25], in their overview of the genetic diversity of *H. capsulatum*, referred to this clade when more *H. capsulatum* isolates were analyzed from *T. brasiliensis* bats.

Based on criteria involving MLST and population structure analyses, after examining the sequences of 234 isolates deposited in different databases, Teixeira et al. [26] proposed that *H. capsulatum* has at least 11 cryptic phylogenetic species, six of which are always concordant (RJ, LAm A1, LAm A2, LAm B1, LAm B2, and BAC1) and reported as new phylogenetic species. According to Teixeira et al. [26], the population structure of the highly diverse LAm A clade has three phylogenetic species (RJ, LAm A1, and LAm A2); besides, although the LAm B phylogenetic species showed low variation compared to other clades [17,20], its analysis suggests the presence of two monophyletic clades (LAm B1 and LAm B2) within LAm B.

Recent information about *H. capsulatum* genetic diversity is based on a precise approach reported by Sepúlveda et al. [27], using whole-genome resequencing data and the phylogenetic analyses of *Histoplasma* isolates from endemic areas of histoplasmosis, mainly from the United States of America (USA). Sepúlveda et al. [27] studied 30 isolates from five phylogeographical clusters (Panama lineage-H81, NAm 1, NAm 2, LAm A, and African) and considering their results, renamed four of them: *H. capsulatum sensu stricto* Darling 1906 (comprising three isolates from the Panama lineage-H81), *H. mississippiense* sp. nov. (comprising 10 isolates from the NAm 1clade); *H. ohiense* sp. nov. (comprising 11 isolates from the NAm 2 clade); and *H. suramericanum* sp. nov. (comprising four isolates from Colombia, previously classified as the LAm A clade). The African phylogenetic species remains without a taxonomic modification in the *H. capsulatum* complex.

The aim of this study was to contribute to the phylogenetic understanding of the *H. capsulatum* complex, by incorporating an important number of isolates from bats, the main wild host of this fungus. We analyzed the sequences of 176 isolates, 30 of which were isolated from wild bats. The *H. capsulatum* phylogeny, based on the GCPSR criterion, was reconstructed using individual locus and concatenated analyses of four protein-coding genes. To do this, we used parsimony, maximum likelihood, and Bayesian inference methods, as well as the coalescence-based methods. A concatenated sequence-types network and nucleotide diversity were also generated. Overall, these analyses provide robust support for the existence of a species-level clade containing seven *H. capsulatum* isolates from bats.

## 2. Materials and Methods

### 2.1. New Histoplasma capsulatum Isolates Studied

The sequences of 42 isolates were analyzed for the first time. These isolates are deposited in the *H. capsulatum* Culture Collection of the Fungal Immunology Laboratory (http://www.wfcc.info/ccinfo/index.php/strain/display/817/fungi/), Department of Microbiology and Parasitology, School of Medicine-UNAM, where they are maintained. This collection is registered in the database of the World Data Centre for Microorganisms as LIH-UNAM WDCM817. The data on bats and clinical *H. capsulatum* isolates used in this study are accessible in the culture collection catalogue, partially published by Rodríguez-Arellanes et al. [31] and in a website (https://www.facmed.unam.mx/histoplas-mex/).

For the isolation of *H. capsulatum* from randomly captured bats, only those species not in danger of extinction were processed, and they were used solely for research purposes. In all cases, national regulations for bat species protection, capture, and processing were strictly complied with, and we adhered to ethical recommendations and to the guidelines published by Gannon, Sikes, and the Animal Care and Use Committee of the American Society of Mammalogists [32]. Bats were processed for fungal isolation in accordance with the Ethics Committee of the School of Medicine, UNAM, following the recommendations of the Animal Care and Use Committee of the UNAM and the Mexican Official Guide (NOM 062-ZOO-1999).

Clinical isolates from Mexico were obtained from different hospitals in the country. Clinical isolates from Argentina and Colombia were donations to our collection by the *INEI-ANLIS-“Dr. Carlos G. Malbrán”* and the *Corporación para Investigaciones Biológicas* institutions, respectively. All clinical isolates were obtained as part of standard care procedures for fungal diagnosis, in hospital microbiology laboratories.

This study was approved by the School of Medicine Research and Ethics Committee (Ref. No. 017/2014).

### 2.2. Histoplasma capsulatum Sequences

Sequences of four individual loci from a total of 176 isolates were analyzed, considering the 42 new isolates here processed (details in Table 1) together with 134 isolates previously reported by Kasuga et al. [20]. Of the 134 isolates studied by Kasuga et al. [20], 17 of them derived from infected bats captured in Mexico. Incomplete sequences of three isolates (EH-325, EH-383, and H190) were omitted from the total 137 isolates studied by Kasuga et al. [20]. Four sequences of *H. capsulatum* reference strains, whose genomes are available at the National Center for Biotechnology Information (https://www.ncbi.nlm.nih.gov/bioproject) were considered in all phylogenetic analyses of the present study: G-217B (accession number PRJNA12653) from the NAm 2 clade; H143 (accession number PRJNA29161) and H88 (accession number PRJNA29163) from the African clade; and G-186A (accession number PRJNA12635) from the Panama lineage. In this study, it is important to remark that the *H. capsulatum* strains named by Kasuga et al. [20] as H8, H81, H82, and H83, here are reported as G-217B, G-184B, G-186A, and G-186B, respectively.

### 2.3. DNA Extraction, PCR, and Sequencing of Histoplasma capsulatum Isolates

DNA extraction was performed on the 42 new isolates, according to Taylor et al. [9]. We processed PCR products of the *H. capsulatum* gene fragments (*arf*, *H-anti*, *ole1*, and *tub1*) as described by Kasuga et al. [20] with minor modifications as per Taylor et al. [21]. Amplicons were sequenced at the High-Throughput Genomics Center (University of Washington, Seattle, WA, USA). DNA sequencing reactions were implemented for both DNA strands and a consensus sequence was generated for each gene fragment using MESQUITE version 3.01 (http://mesquiteproject.org) and Chromas Lite version 2.1.1 (http://technelysium.com.au/. Sequences of the 42 new *H. capsulatum* isolates were deposited in the GenBank (see Table 1). The sequences from Kasuga et al. [20] are available on the TreeBASE database (study ID S1063).

### 2.4. Histoplasma capsulatum Sequence Alignments and BLASTn Analyses

The sequences of 176 isolates were assembled and aligned manually using MESQUITE (http://mesquiteproject.org). A concatenated matrix containing the four gene fragments studied was used for phylogenetic reconstruction.

A BLASTn analysis [33] was conducted with the complete genes reported in the GenBank (accession numbers: L25117.1, U20346.1, X85962.1, and M28358.1 for *arf*, *H-anti*, *ole1*, and *tub1*, respectively) for the G-217B strain (American Type Culture Collection-26032) from Louisiana/USA, which is considered the most representative strain of the NAm 2 phylogenetic species.

### 2.5. Congruence Analysis

Congruence of the four gene genealogies was evaluated with the incongruence length difference (ILD) test developed by Farris et al. [34] and implemented in PAUP* version 4. 2003 as the partition homogeneity test (http://paup.csit.fsu.edu/downl.html). For each test, uninformative characters were excluded, and the sum of tree lengths of the actual partition was compared to the sum of tree lengths of 1000 randomly assigned partitions, where the null hypothesis is that the *arf*, *H-anti*, *ole1*, and *tub1* partitions are congruent (the sequences are drawn from a single, homogeneous group of characters). The percent of instances where the sum of the tree lengths of each random partition exceeded that of the true partition was used to detect incongruence between data sets.

### 2.6. Phylogenetic Reconstruction

The four gene regions were subjected to two-way comparisons in all possible combinations and analyzed by different methods. (1) Parsimony analysis was performed with TNT version 1.1 [35] using a random starting tree with 1000 ratchet iterations [36]; all characters were treated as unordered and assigned equal weights. (2) Probabilistic analyses were performed with maximum likelihood (ML) and Bayesian inference (BI). ML analysis was conducted in RAxMLGUI version 1.31 [37] using the general time reversible (GTR) substitution model with a gamma distribution. BI was performed in MrBayes version 3.2 [38] using four chains with a total of 100,000,000 generations and sampling trees every 10,000 generations. Convergence of the chains was evaluated with the effective sample size (ESS) values and corroborated with Tracer version 1.6 (http://beast.bio.ed.ac.uk/Tracer). Both probabilistic analyses were implemented in jModeltest version 2.1.4 [39]. Based on the results of the Bayesian information criterion of jModeltest, the substitution models considered for each partition were K80 (*H-anti*), K80 + G (*arf* and *tub1*), and K80 + I (*ole1*).

Bootstrap (bt) values for parsimony and ML analyses were based on the heuristic search of 1000 replicates, using tree-bisection-reconnection. For the BI, the maximum clade credibility tree was selected with a posterior probability (pp) limit of 0.95, using TreeAnnotator version 1.8.2, implemented with *BEAST [40]. Unrooted trees were constructed using concatenated and individual sequence alignments. In special cases, rooted trees were generated with *Blastomyces dermatitidis* as an outgroup, using the sequences available in the GenBank database (accession numbers: *arf*-XM002628904.1; *ole1*-XM002625814.1; and *tub1*-JN562337.1). In the concatenated analyses, the *H-anti* gene fragment was considered as missing data.

### 2.7. Coalescence Analysis

A coalescence-based analysis was conducted using the *BEAST method, which was implemented in BEAST version 1.8.2 [40,41]. An XML file was generated for the alignments of the four loci using BEAUti version 1.8.2 [41]. The K80 substitution model with empirical base frequencies was applied to the four loci, and the gamma distribution was included for *arf* and *tub1*, whereas invariant sites were included in the model for *ole1*. The remaining parameters used in the *BEAST coalescence analysis were the same as those used in the BI phylogenetic analysis. The final run of the coalescence analysis assumed a strict molecular clock based on the results of the stepping-stone and marginal likelihoods methods implemented in MrBayes software version 3.2 [38,41], which tested strict clock vs. no clock or strict clock vs. some sort of relaxed clock. Here, we used the nucleotide substitution rates reported by Kasuga et al. [20], which were estimated considering two divergence times from Eurotiomycetes, 127.8 million years ago for *Histoplasma* and 31.8 million years ago for *Blastomyces*; *arf*: 0.86 × 10^−9^, *H-anti*: 1.17 × 10^−9^, *ole1*: 0.87 × 10^−9^, and *tub1*: 1.63 × 10^−9^ substitutions/site/year.

### 2.8. Concatenated Sequence-Types (CSTs) Network

The concatenated matrix of four nuclear genes was used to generate an unrooted network constructed by the median-joining algorithm [42] with Network version 4.613 (www.fluxus-engineering.com).

### 2.9. Nucleotide Diversity (π)

Estimation of intraspecific π values for *arf*, *H-anti*, *ole1*, and *tub1* gene fragments of the *H. capsulatum* isolates studied was performed on the concatenated alignment using DnaSP version 5.10 [43].

## 3. Results

Of the 42 newly reported isolates, 13 were obtained from naturally infected bats captured in different Mexican regions and 29 were isolated from human clinical samples (8 from Argentina, 11 from Colombia, 2 from Guatemala, and 8 from Mexico) (see Table 1).

### 3.1. Histoplasma capsulatum BLASTn Analysis

High similarity (95–99%) was found by BLASTn among all the sequences studied, when compared with the sequences of the four complete genes (*arf*, *H-anti*, *ole1*, and *tub1*) of the G-217B reference strain.

### 3.2. Congruence Analysis

The concatenated matrix of the four gene fragments had a total of 1538 nucleotides (nt), of which 321 sites were variable and 226 were parsimony informative (Table 2). The length of each gene fragment studied was as follows: *arf*-457 nt, *H-anti*-397 nt, *ole1*-414 nt, and *tub1*-270 nt.

The ILD test found no significant heterogeneity among the four gene genealogies (Table 3).

### 3.3. Phylogenetic Reconstruction

**Figure 1:**
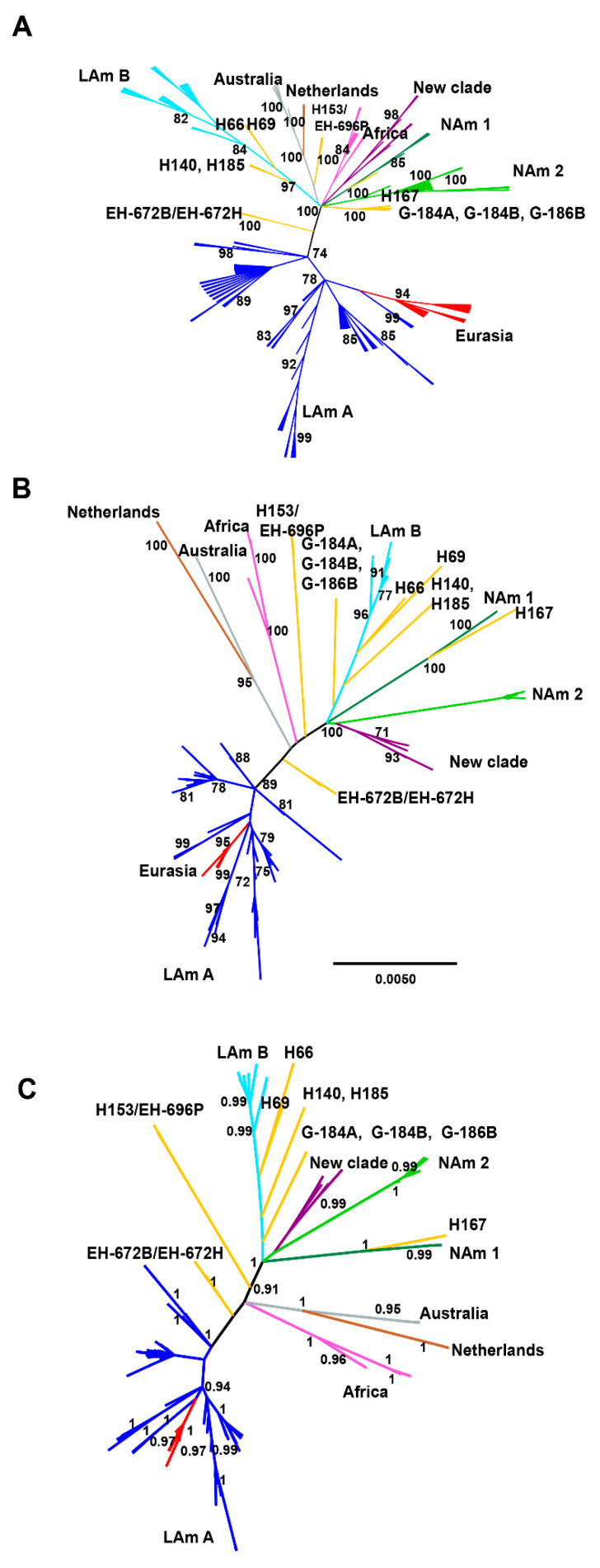
Unrooted phylogenetic trees of *H. capsulatum* isolates generated by different inference methods. The analyses were performed with a concatenated matrix of 1538-nt constructed with four gene fragments (*arf*, *H-anti*, *ole1*, and *tub1*). (**A**) Parsimony strict consensus tree with a bt ≥ 70%; (**B**) maximum likelihood tree with a bt ≥ 70%; (**C**) Bayesian inference maximum clade credibility tree was selected with a pp limit of 0.95. The support values of bt and pp are indicated on their corresponding tree nodes (details under Materials and Methods).

In all phylogenetic analyses of the concatenated alignments, using the sequences of the 176 *H. capsulatum* isolates studied, the eight clades described by Kasuga et al. [20] and the LAm A1, LAm A2, LAm B1, and BAC1 clades named by Teixeira et al. [26] were recognized (see Figure 1A–C).

Due to the ML and BI trees have similar topologies in the individual analysis for the alignments of each locus studied; they were represented as BI trees. The support for each branch was included as bt values for ML/pp values for BI analyses (see Supplementary Material, Figures S1–S4).

The parsimony analysis of the concatenated alignment resulted in eight most parsimonious trees, a tree length of 514 steps, a consistency index of 0.654, and a retention index of 0.934 (Figure 1A).

According to our data, most of the new *H. capsulatum* isolates analyzed match with LAm A phylogenetic species described by Kasuga et al. [20]. In addition, 28 of these new isolates clustered together with some isolates previously classified by Teixeira et al. [26] as belonging to the phylogenetic species LAm A1, LAm A2, LAm B1, and BAC1, with the exception of isolates 1558 and 1739 from Argentina, DS and LF from Colombia, as well as EH-323, EH-324, EH-326, EH-327; EH-355, EH-356, EH-357, EH-672B, EH-672H, and EH-696 from Mexico, which formed different independent groups (see Table 1).

In agreement with the results by Kasuga et al. [20], the present data showed that LAm A forms a clade that also contains the Eurasian isolates. Furthermore, LAm A is connected to almost all the other isolates by a long and well-supported internal branch. A branch leading to the two isolates, EH-672B and EH-672H, is always attached to this long internal branch in the concatenated analyses (Figure 1A–C).

Unrooted phylogenetic trees (Figure 1A–C) had similar topologies and showed slight differences between parsimony (Figure 1A) and BI (Figure 1C) trees.

In the present analyses, the EH-315 isolate obtained from a *M. megalophylla* bat captured in Mexico and classified as a lone lineage by Kasuga et al. [20], forms a particular clade (bt > 70% and pp > 0.95), together with six *H. capsulatum* isolates (EH-384I, EH-384P, EH-655P, EH-658H, EH-670B, and EH-670H) obtained from *T. brasiliensis* bats captured in different regions of Mexico (Figure 1A–C); although isolates EH-384I, EH-384P, and EH-315 were slightly divergent. This clade forms a polytomy with several others in the parsimony tree (Figure 1A), including LAm B, Netherlands−Australian, African, and NAm 1, as well as NAm 2; however, notably in the ML and BI analyses, this clade is the sister group of NAm 2 (Figure 1B,C). Given its relationship to NAm 2, we propose naming this new clade formed here with seven *H. capsulatum* isolates as NAm 3, highlighting that this clade incorporated three *H. capsulatum* isolates that had been previously described by Taylor et al. [21] and later considered as a new phylogenetic species denominated BAC1 by Teixeira et al. [26].

Another of the new *H. capsulatum* isolates, EH-696P, obtained from a *T. brasiliensis* bat captured in the state of Nuevo León in northwestern Mexico had similar sequences to the isolate H153 (100% bt; 1.0 pp) from a Brazilian patient, which had formerly been classified as a lone lineage by Kasuga et al. [20] and Teixeira et al. [26].

Here, we also found a new lone lineage composed of *H. capsulatum* isolates EH-672B and EH-672H, both obtained from a *T. brasiliensis* bat captured in the state of Hidalgo, Mexico. This lone lineage had high support values in parsimony (bt = 100, Figure 1A) and BI (pp = 1, Figure 1C) analyses, although a bt < 70% was found in the ML analysis. The relationships of the EH-672B/EH-672H isolates to all other clades and lone lineages are unclear in the analyses using the concatenated matrix (Figure 1A–C), and in *H-anti*, *ole1*, and *tub1* individual trees, these isolates are included in different clades (see Supplementary Material, Figures S2–S4); regarding the *arf* gene, the amplified fragments generated for these isolates showed a lower query cover than the compared reference sequences of the GenBank, affecting their analyses.

All phylogenetic rooted trees were constructed using *B. dermatitidis* sequences as an outgroup. The results showed similar topologies to those for unrooted trees, although the branch between the outgroup and the *H. capsulatum* isolates was longer in the individual gene trees (Supplementary Material, Figures S5–S8).

### 3.4. Coalescence Analysis

**Figure 2:**
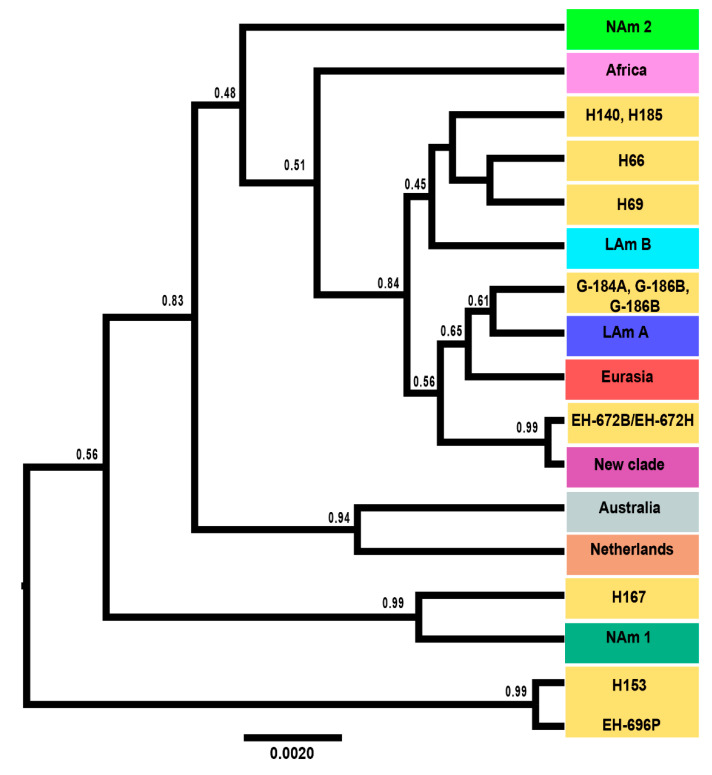
Species tree generated by coalescent-based species delimitation methods of the *H. capsulatum* complex. Maximum-clade-credibility tree of the concatenated gene fragments *arf*, *H-anti*, *ole1*, and *tub1* was selected from the *BEAST analysis, using a strict molecular clock (see Materials and Methods). The values of pp are indicated on their corresponding branches of the tree nodes.

Convergence among runs was found in the *BEAST analysis using a strict molecular clock; the ESS values were >200. The *BEAST analysis was also performed using a relaxed molecular clock, and the same topology was recovered. However, the topology of the resulting species tree (Figure 2) was different from those of all phylogenetic analyses. It showed that the Nam 3 clade and most of the lone lineages were closely related with the Latin American and the Eurasian clades (0.84 pp) described by Kasuga et al. [20], except for a lone lineage H167 from Argentina that was sister to NAm 1 (0.99 pp) and for the lone lineage formed by H153 and EH-696P isolates (0.99 pp), which was sister to all other phylogenetic species. The Australian and Netherlands clades grouped together (0.94 pp), as found in the MLST analyses performed by Kasuga et al. [20]. In the coalescence analysis (Figure 2), the EH-672B/EH-672H lone lineage is a close relative of the newly-named NAm 3 clade (0.99 pp).

### 3.5. Concatenated Sequence-Types (CSTs) Network

**Figure 3:**
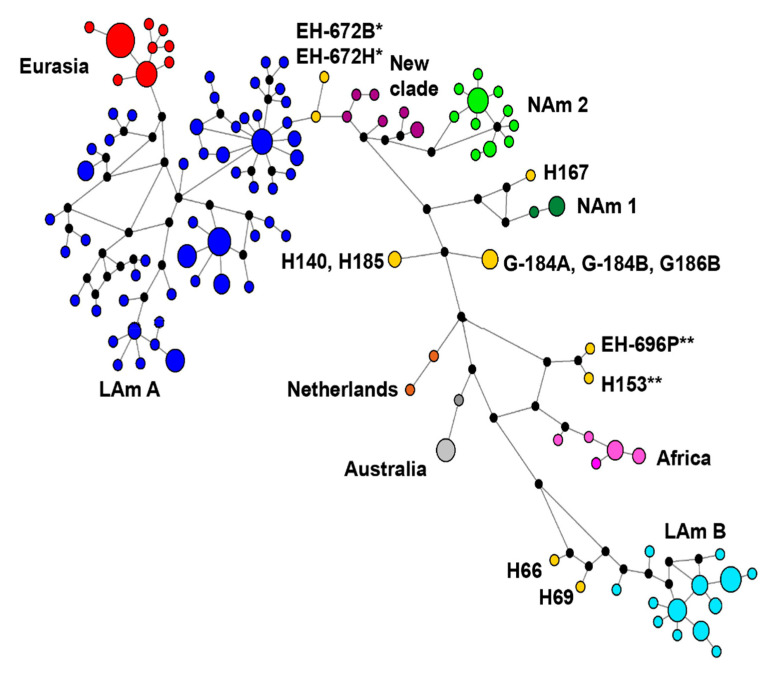
Dispersion of the *H. capsulatum* complex associated with concatenated sequence-types (CSTs) from the 176 isolates studied. A total of 110 CSTs were generated using a median-joining network based on the analysis of the 1538-nt alignment of four concatenated gene fragments. The branch lengths are proportional to the number of substitutions, and the relative sizes of circles are proportional to their corresponding CST frequencies. Each node (black circle) indicates a hypothetical missing CST. The CST colors correspond to different *H. capsulatum* clades and the lone lineages are in yellow.

Concatenated sequence-types (CSTs) network analysis found a total of 110 distinctive CSTs from the four loci concatenated matrix of the 176 *H. capsulatum* isolates (Figure 3). In the CSTs analysis it was possible to confirm that the LAm A clade was the most genetically differentiated (52 CSTs), followed by the LAm B (13 CSTs), NAm 2 (11 CSTs), Eurasian (9 CSTs), NAm 3 (6 CSTs), and African (5 CSTs) clades. The least genetically differentiated clades were NAm 1 (2 CSTs), Netherlands (2 CSTs), and Australian (2 CSTs). Of the 110 CSTs, nine occurred as lone lineages (Figure 3).

In terms of distance among CSTs, the LAm A and the LAm B clades were the most distant, the Eurasian CSTs emerged from LAm A, the new EH-672B/EH-672H lone lineage was connected to LAm A, and the NAm 3 clade was associated to NAm 2. The African, Netherlands and Australian clades were closely grouped (Figure 3). These last relationships can also be seen in all phylogenetic analyses using a concatenated matrix (Figure 1A–C).

### 3.6. Nucleotide Diversity (π)

In regard to genetic diversity, intraspecific π values for clades ranged from the most diverse (LAm A = 0.00835) to the least diverse (Australian = 0.00027). The NAm 3 clade had a π value of 0.0061 rather the African clade (π = 0.00622). The nucleotide diversity of the lone lineages revealed π values in the range of 0.00528 (EH-672B/EH-672H) to 0.00342 (H153/EH-696P).

## 4. Discussion

The role of bats in spreading *H. capsulatum* in the environment was proposed many years ago, particularly by Hoff and Bigler [7]; however, the relationship between the behavior of bats and *H. capsulatum* ecology remains ambiguous, especially the potential connection of their movements and migrations with this pathogen’s dispersion in nature [8].

Regarding the bat−*Histoplasma* interplay, the first important finding to this binomial relationship concerns a lone lineage (EH-315) described by Kasuga et al. [20], which was formed by one *H. capsulatum* isolate from an infected *M. megalophylla* bat captured in Mexico. According to our novel data and major sequence analyses, a cluster comprising six *H. capsulatum* isolates (EH-384I, EH-384P, EH-655P, EH-658H, EH-670B, and EH-670H) associated with *T. brasiliensis* bats, together with the EH-315 isolate, formed a NAm 3 clade that was supported in the phylogenetic reconstruction analyses with values of bt > 70% and pp > 0.95 and was well defined in the CSTs network. Thus, it is reasonable to consider the NAm 3 clade as a phylogenetic species, based on the GCPSR concept recognized by Taylor et al. [29] and Mayden [44], in agreement with Kasuga et al. [20] and Teixeira et al. [26]. The two wild bat species from which these *H. capsulatum* isolates were recovered are colonial and share some attributes, such as insectivorous feeding, habitats, and migratory behavior [45,46].

Considering the inclusion of an important number of new *H. capsulatum* isolates from different sources, our results support the high genetic diversity of this pathogen by using multifaceted methods for phylogenetic and species tree inference. The present study confirms the earlier molecular phylogenetic relationships of the *H. capsulatum* species complex, reported by Kasuga et al. [20] and Teixeira et al. [26], and it replaces the BAC1 clade (with only three isolates) proposed by Teixeira [26] with the NAm 3 clade (containing seven isolates), which revealed itself to be more closely related to the NAm 2 phylogenetic species, by concatenated sequence analyses and CSTs network findings.

**Figure 4:**
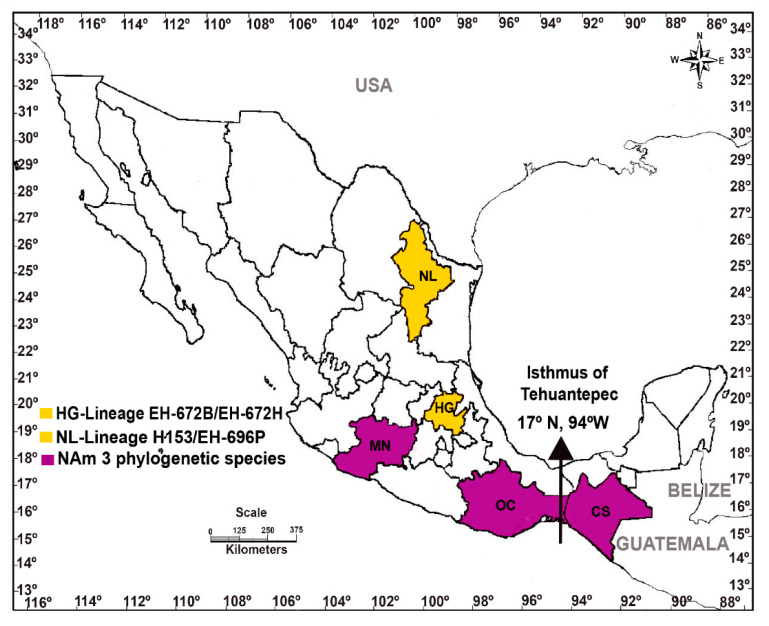
Distribution of *H. capsulatum* isolated from the migratory bat *T. brasiliensis* in Mexico. Geographically, Mexico is in North and Central America, and the boundary of both regions is delimited by the Isthmus of Tehuantepec (arrowhead). *T. brasiliensis* bats were captured in different states of Mexico, which are distributed in either the Northern or Central American regions of the Mexican territory. CS: Chiapas; HG: Hidalgo; MN: Michoacán; NL: Nuevo León; OC: Oaxaca.

*Tadarida brasiliensis* was the major bat species associated with *H. capsulatum* isolates from the NAm 3 phylogenetic species, the lone lineage EH-672B/EH-672H, and the EH-696P isolate that clustered together with the lone lineage H153, previously described by Kasuga et al. [20]. This bat species was captured in different states of the Mexican territory included in North or Central America (see Figure 4). In the past, Taylor et al. [9] described that the GACG(GA)11GA haplotype of the (GA)n microsatellite and its flanking regions is associated with nine *H. capsulatum* isolates from *T. brasiliensis* captured in the southern region of Mexico (Chiapas and Oaxaca states); of these nine *H. capsulatum* isolates, six (EH-384I, EH-384H, EH-655P, EH-658H, EH-670B, and EH-670H) were classified here as belonging to the NAm 3 phylogenetic species. Based on these findings, it is reasonable to consider that gene flow mechanisms could displace *H. capsulatum* genetic patterns in the environment, mainly associated with special wild hosts. Particularly, according to our findings, it is possible to expect that *T. brasiliensis* has at least three different migratory routes in the Mexican territory (see Figure 4), based on the genetic diversity of the *H. capsulatum* isolates recovered from this bat species. Interestingly, the subspecies *T. brasiliensis mexicana* has a migratory route that extends from the southwestern regions of the USA to the northern and central-southeastern regions of Mexico [47]. Thus, the geographic distribution of *H. capsulatum* could be related to the migratory behavior of infected bats, considering their possible evolutionary history with this pathogen in shared natural habitats [9,20,21,26].

In regard to the motivating data published by Sepúlveda et al. [27], who by using phylogenomic species recognition, which presented the *Histoplasma* American phylogenetic species as *H. capsulatum sensu stricto*, *H. mississippiense*, *H. ohiense*, and *H. suramericanum*, it is noteworthy that none of the new *H. capsulatum* isolates analyzed here were compatible with the phylogenetic species reported by Sepúlveda et al. [27], probably because we followed different methodologies for species recognition.

With respect to the congruence analysis, in contrast to the report by Kasuga et al. [17], we found no evidence of incongruence among individual gene trees by the ILD test [34] (see Table 3). This discrepancy may be due to our inclusion of additional isolates, which may increase support for branches among species and reduce it within species. Besides, even if incongruence is detected, it may not provide a conclusive demonstration that concatenation of data produces phylogenetic error [48].

The present results also improve our understanding of Latin American *H. capsulatum* phylogeographic distribution. Based on phylogenetic reconstruction and coalescence analyses, it was found that most of the *H. capsulatum* isolates from Mexico and Colombia here studied are in the LAm A clade. However, two new *H. capsulatum* isolates from Colombia and all the new isolates from Argentina detailed in Table 1 were shown to belong to the LAm B clade according to Kasuga et al. [20] or LAm B1 considering the modified classification of Teixeira et al. [26]. In consequence, there may be an association between Latin American clades and geography in South America.

In our study, unrooted phylogenetic trees were primarily generated; additionally, rooted trees were also constructed using *B. dermatitidis* as an outgroup and only considering the accessible sequences for *arf, ole1,* and *tub1*. It is important to comment that *H-anti* was not used in the construction of the rooted trees because its sequences were not available for *B. dermatitidis* or any fungus that could be used as an outgroup, such as *B. parvus*, *Paracoccidioides brasiliensis*, *P. lutzii* or *Emmonsia crescens*. Overall, the topologies of rooted trees agreed with each clade generated by unrooted trees, as mentioned in the results section. Slight discrepancies in the topologies of unrooted trees, involving short internal branches of some clades, could be explained by the different phylogenetic analyses used, which is consistent with an early and short period of *H. capsulatum* radiation.

The *H. capsulatum* isolates from the NAm 3 phylogenetic species share a high similarity among their CSTs (see Figure 3) and in their phylogenetic reconstruction (see Figure 1A–C). Our data also highlight that NAm 3 has strong support in the four unrooted individual gene trees and does not contain other taxa.

The CST network is a general evolutionary representation that infers ancestral types, variants, and estimates dating and provides strong support to investigate the relationships among all *H. capsulatum* isolates studied. Based on the number of CSTs found for each clade, LAm A was the most diverse and the best sampled. Besides, NAm 3 was also diverse, irrespective of the number of isolates analyzed.

The coalescence analysis using the *BEAST method is a useful tool for inferring relationships among groups of isolates, but in a few cases, it revealed conflicting relationships when compared with other phylogenetic and network methods, which have somewhat lower support. However, the lower bt and pp values shown in this coalescent-based method are possibly due to the lack of more molecular data. This occurred in the present analyses as well as in other reports [20].

Regarding the independent lineages previously described by Kasuga et al. [20], the present results also support a close relationship between the lone lineages H81 (G-184B), H66, and H69 and the LAm B clade (see Figure 1A–C) even though branch support is low. Considering the results reported by Kasuga et al. [20], the discrepancy in branch support reported here could be explained by the use of different methods that often give contrasting results for the same organism, as sustained by Sites and Marshall [49]. In addition, the sister relationship between the Brazilian H153 and the Mexican EH-696P isolates is well supported in the species tree as a lone lineage, although the phylogenetic position of this lineage with respect to other *H. capsulatum* isolates remains unclear. This latter issue should be investigated further with more loci.

Intraspecific π values were obtained for *H. capsulatum* isolates only to detect gene diversity among isolates of the same clade or the same lone lineage, whereas determination of interspecific π values were unnecessary because divergences among clades and lineages were well sustained by all phylogenetic analyses reported here. Our data indicate that the intraspecific variation in NAm 3 (π = 0.0061), LAm A (π = 0.00835), and African (π = 0.00622) clades was homogeneous. Finally, considering the number of CSTs (6 CSTs) and the π value (π = 0.0061), the existence of a different evolutionary line supports the new phylogenetic species NAm 3.

## 5. Conclusions

The present information about the interaction between the fungal pathogen *H. capsulatum* and one of its most important wild hosts is unique. We analyzed sequences of nine *H. capsulatum* isolates from *T. brasiliensis*: six (EH-384I, EH-384P, EH-655P, EH-658H, EH-670B, and EH-670H) belong to the NAm 3 phylogenetic species, while the other three (EH-672B, EH-672H, and EH-696P) are proposed as lone lineages. Concatenated sequence analyses and the CSTs network support these findings in the *H. capsulatum* complex. Interestingly, the NAm 3 phylogenetic species and the EH-672B/EH-672H lineage reported here are known only from naturally infected bats captured in Mexico, which may suggest that specific mammals are susceptible to particular genotypes of *H. capsulatum*, a possibility that warrants future comparison of their genomes. Thus, the detection of fungal genotypes associated with geographical patterns in infected bats randomly captured in the environment could contribute as a molecular biomarker to monitor the movements and migrations of bats and also to generate an epidemiological map of *H. capsulatum*, according to its distribution in nature. Furthermore, our results highlight the importance of histoplasmosis as a global health issue, including the unusual aspects of the pathogen *H. capsulatum* involving naturally infected bats.

## Figures and Tables

**Table 1:** Data about the new *H. capsulatum* isolates reported in the present study.

Isolate Related		Phylogenetic-	Origin	GenBank (Accession Numbers)			
Acronym	Source	Species/Lineage ^a^		*arf*	*H-anti*	*ole1*	*tub1*
1558	Human	LAm B (*)	AR	KT601344	KT601418	KT601381	KT601463
1739	Human	LAm B (*)	AR	KT601345	KT601419	KT601382	KT601464
92590	Human	LAm B (LAm B1)	AR	KT601346	KT601420	KT601383	KT601465
951814	Human	LAm B (LAm B1)	AR	KT601347	KT601421	KT601384	KT601466
993444	Human	LAm B (LAm B1)	AR	KT601348	KT601423	KT601385	KT601467
993445	Human	LAm B (LAm B1)	AR	KT601349	KT601424	KT601386	KT601468
993446	Human	LAm B (LAm B1)	AR	KT601350	KT601425	KT601387	KT601469
993267	Human	LAm B (LAm B1)	AR	KT601351	KT601422	KT601388	KT601470
AP	Human	LAm A (LAm A1)	CO	KT601352	KT601427	KT601389	KT601471
DS	Human	LAm A (*)	CO	KT601353	KT601428	KT601390	KT601472
GeM	Human	LAm A (LAm A1)	CO	KT601354	KT601445	KT601391	KT601473
GLi	Human	LAm A (LAm A2)	CO	KT601355	KT601446	KT601392	KT601474
Hz	Human	LAm B (LAm B1)	CO	KT601356	KT601449	KT601393	KT601475
JG	Human	LAm B (LAm B1)	CO	KT601357	KT601450	KT601394	KT601476
LA	Human	LAm A (LAm A2)	CO	KT601358	KT601451	KT601395	KT601477
LF	Human	LAm A (*)	CO	KT601359	KT601452	KT601396	KT601478
MZ2	Human	LAm A (LAm A2)	CO	KT601360	KT601426	KT601397	KT601479
RG	Human	LAm A (LAm A1)	CO	KT601361	KT601453	KT601398	KT601480
WCh	Human	LAm A (LAm A1)	CO	KT601362	KT601454	KT601399	KT601481
H.1.02.W	Human	LAm A (LAm A2)	GT	KT601363	KT601447	KT601400	KT601482
H.1.12.96	Human	LAm A (LAm A2)	GT	KT601364	KT601448	KT601401	KT601483
EH-323	Human	LAm A (*)	MX	KT601365	KT601429	KT601402	KT601484
EH-324	Human	LAm A (*)	MX	KT601366	KT601430	KT601403	KT601485
EH-326	Human	LAm A (*)	MX	KT601367	KT601431	KT601404	KT601486
EH-327	Human	LAm A (*)	MX	KT601368	KT601432	KT601405	KT601487
EH-328	Human	LAm A (LAm A1)	MX	KT601369	KT601433	KT601406	KT601488
EH-355	Human	LAm A (*)	MX	KT601370	KT601434	KT601407	KT601489
EH-356	Human	LAm A (*)	MX	KT601371	KT601435	KT601408	KT601490
EH-357	Human	LAm A (*)	MX	KT601372	KT601436	KT601409	KT601491
EH-383I ^b^	*L. nivalis*	LAm A (LAm A1)	MX	AF495619	AF495620	AF495621	F495622
EH-383P ^b^	*L. nivalis*	LAm A (LAm A1)	MX	AF495623	AF495624	AF495625	AF495626
EH-384I ^b^	*T. brasiliensis*	NAm 3 (BAC1)	MX	AF495627	AF495628	AF495629	AF495630
EH-384P ^b^	*T. brasiliensis*	NAm 3 (BAC1)	MX	AF495631	AF495632	AF495633	AF495634
EH-408H ^b^	*L. nivalis*	LAm A (LAm A1)	MX	AF495644	AF495643	AF495645	AF495646
EH-449B	*L. nivalis*	LAm A (LAm A1	MX	KT601373	KT601437	KT601410	KT601455
EH-655P	*T. brasiliensis*	NAm 3 (BAC1)	MX	KT601374	KT601438	KT601411	KT601458
EH-658H	*T. brasiliensis*	NAm 3 (BAC1)	MX	KT601375	KT601439	KT601412	KT601459
EH-670B	*T. brasiliensis*	NAm 3 (BAC1)	MX	KT601376	KT601440	KT601414	KT601460
EH-670H	*T. brasiliensis*	NAm 3 (BAC1)	MX	KT601377	KT601441	KT601415	KT601461
EH-672B	*T. brasiliensis*	NAm 3 (*)	MX	KT601378	KT601442	KT601413	KT601456
EH-672H	*T. brasiliensis*	NAm 3 (*)	MX	KT601379	KT601443	KT601416	KT601457
EH-696P	*T. brasiliensis*	H153-lineage (*)	MX	KT601380	KT601444	KT601417	KT601462

^a^ Phylogenetic species of the new isolates studied, based on the classification as Kasuga et al. [20] and, in parenthesis, as Teixeira et al. [26]. ^b^ Isolates previously studied by Taylor et al. [21], without phylogenetic classification. (*) *H. capsulatum* isolates not included previously in any phylogenetic species. Bat species: *L. nivalis* = *Leptonycteris nivalis*; *T. brasiliensis* = *Tadarida brasiliensis*. NAm 3: North American 3. AR: Argentina; CO: Colombia; GT: Guatemala; MX: Mexico.

**Table 2:** Genetic diversity of each gene fragment analyzed.

Gene Fragments			Nucleotide Sites		
	Size (nt)	^a^ Start/End (nt)	Variable		Parsimonious
				Informative	Uninformative
*arf*	457	415/871	70	44	26
*H-anti*	397	394/789	91	59	32
*ole1*	414	37/450	69	49	20
*tub1*	270	590/862	91	74	17
Total	1538		321	226	95

^a^ Reference data came from each complete gene of the G-217B *H. capsulatum* strain deposited in GenBank (see Materials and Methods).

**Table 3:** Data for the incongruence length difference test using the sequences of the four genes studied.

Sum of Tree Lengths			
Partition	Original Partition	Range of Replicates	*p* Value
Four genes	1243	1243–1242	0.997
*arf* vs. *H-anti*	161	161–0	1
*arf* vs. *ole1*	156	156–157	0.941
*arf* vs. *tub1*	193	193–194	0.997
*H-anti* vs. *ole1*	168	168–169	0.748
*H-anti* vs. *tub1*	217	217–0	1
*ole1* vs. *tub1*	202	202–0	1

The ILD test was performed with the 176 H. capsulatum isolates.

## Data Availability

Not applicable.

## Supplementary Materials

The following are available online at https://www.mdpi.com/article/10.3390/jof7070529/s1, Figure S1: Unrooted phylogenetic tree of the *arf* gene fragment for *H. capsulatum* isolates. The Bayesian inference maximum clade credibility tree was selected with a pp limit of 0.95. The bt and pp values are indicated on their corresponding tree nodes (details under Materials and Methods). Supporting values are indicated as follows: bt/pp in maximum likelihood/Bayesian inference, respectively. Figure S2: Unrooted phylogenetic tree of the *H-anti* gene fragment for *H. capsulatum* isolates. The Bayesian inference maximum clade credibility tree was selected with a pp limit of 0.95. The bt and pp values are indicated on their corresponding tree nodes (details under Materials and Methods). Supporting values are indicated as follows: bt/pp in maximum likelihood/Bayesian inference, respectively. Figure S3: Unrooted phylogenetic tree of the *ole1* gene fragment for *H. capsulatum* isolates. The Bayesian inference maximum clade credibility tree was selected with a pp limit of 0.95. The bt and pp values are indicated on their corresponding tree nodes (details under Materials and Methods). Supporting values are indicated as follows: bt/pp in maximum likelihood/Bayesian inference, respectively. Figure S4: Unrooted phylogenetic tree of the *tub1* gene fragment for *H. capsulatum* isolates. The Bayesian inference maximum clade credibility tree was selected with a pp limit of 0.95. The bt and pp values are indicated on their corresponding tree nodes (details under Materials and Methods). Supporting values are indicated as follows: bt/pp in maximum likelihood/Bayesian inference, respectively. Figure S5: Rooted phylogenetic tree of the *arf* gene fragment for *H. capsulatum* isolates. A Bayesian inference maximum clade credibility tree was selected with a pp limit of 0.95. The pp values are indicated on their corresponding tree nodes. A *B. dermatitidis* sequence was used as an outgroup (see Materials and Methods). Figure S6: Rooted phylogenetic tree of the *ole1* gene fragment for *H. capsulatum* isolates. A Bayesian inference maximum clade credibility tree was selected with a pp limit of 0.95. The pp values are indicated on their corresponding tree nodes. A *B. dermatitidis* sequence was used as an outgroup (see Materials and Methods). Figure S7: Rooted phylogenetic tree of the *tub1* gene fragment for *H. capsulatum* isolates. A Bayesian inference maximum clade credibility tree was selected with a pp limit of 0.95. The pp values are indicated on their corresponding tree nodes. A *B. dermatitidis* sequence was used as an outgroup (see Materials and Methods). Figure S8: Rooted phylogenetic tree of the concatenated *arf*, *ole1*, and *tub1*genes of *H. capsulatum* isolates generated by a Bayesian inference method. Maximum-clade-credibility tree was constructed with a concatenated matrix with three gene fragments. The pp ≥ 0.95 values are indicated on their corresponding branches of the tree nodes. *B. dermatitidis* sequences of the three gene fragments available in the GenBank were used as an outgroup.

## References

[1] Taylor M.L., Chávez-Tapia C.B., Vargas-Yañez R., Rodríguez-Arellanes G., Peña-Sandoval G.R., Toriello C., Pérez A., Reyes-Montes M.R. (1999). Environmental conditions favoring bat infection with *Histoplasma capsulatum* in Mexican shelters. Am. J. Trop. Med. Hyg..

[2] Canteros C.E., Iachini R.H., Rivas M.C., Vaccaro O., Madariaga J., Galarza R., Snaiderman L., Martínez M., Paladino M., Cicuttin G. (2005). Primer aislamiento de *Histoplasma capsulatum* de murciélago urbano *Eumops bonariensis*. Rev. Argent. Microbiol..

[3] González-González A.E., Aliouat-Denis C.M., Carreto-Binaghi L.E., Ramírez J.A., Rodríguez-Arellanes G., Demanche C., Chabé M., Aliouat E.M., Dei-Cas E., Taylor M.L. (2012). An *Hcp100* gene fragment reveals *Histoplasma capsulatum* presence in lungs of *Tadarida brasiliensis* migratory bats. Epidemiol. Infect..

[4] González-González A.E., Ramírez J.A., Aliouat-Denis C.M., Demanche C., Aliouat E.M., Dei-Cas E., Chabé M., Taylor M.L. (2013). Molecular detection of *Histoplasma capsulatum* in the lung of a free-ranging common Noctule (*Nyctalus noctula*) from France using the *Hcp100* gene. J. Zoo Wildl. Med..

[5] González-González A.E., Aliouat-Denis C.M., Ramírez-Bárcenas J.A., Demanche C., Pottier M., Carreto-Binaghi L.E., Akbar H., Derouiche S., Chabé M., Aliouat E.M. (2014). *Histoplasma capsulatum* and *Pneumocystis* spp. co-infection in wild bats from Argentina, French Guyana, and Mexico. BMC Microbiol..

[6] Suárez-Alvarez R.O., Sahaza J.H., Berzunza-Cruz M., Becker I., Curiel-Quesada E., Pérez-Torres A., Reyes-Montes M.R., Taylor M.L. (2019). Dimorphism and dissemination of *Histoplasma capsulatum* in the upper respiratory tract after intranasal infection of bats and mice with mycelial propagules. Am. J. Trop. Med. Hyg..

[7] Hoff G.L., Bigler W.J. (1981). The role of bats in the propagation and spread of histoplasmosis: A review. J. Wildl. Dis..

[8] Taylor M.L., Chávez-Tapia C.B., Reyes-Montes M.R. (2000). Molecular typing of *Histoplasma capsulatum* isolated from infected bats, captured in Mexico. Fungal Genet. Biol..

[9] Taylor M.L., Hernández-García L., Estrada-Bárcenas D.A., Salas-Lizana R., Zancopé-Oliveira R.M., García de la Cruz S., Galvão-Dias M.A., Curiel-Quesada E., Canteros C.E., Bojórquez-Torres G. (2012). Genetic diversity of microsatellite (GA)_n_ and their flanking regions of *Histoplasma capsulatum* isolated from bats captured in three Latin-American countries. Fungal Biol..

[10] Vincent R.D., Goewert R., Goldman W.E., Kobayashi G.S., Lambowitz A.M., Medoff G. (1986). Classification of *Histoplasma capsulatum* isolates by restriction fragment polymorphisms. J. Bacteriol..

[11] Spitzer E.D., Lasker B.A., Travis S.J., Kobayashi G.S., Medoff G. (1989). Use of mitochondrial and ribosomal DNA polymorphisms to classify clinical and soil isolates of *Histoplasma capsulatum*. Infect. Immun..

[12] Spitzer E.D., Keath E.J., Travis S.J., Painter A.A., Kobayashi G.S., Medoff G. (1990). Temperature-sensitive variants of *Histoplasma capsulatum* isolated from patients with acquired immunodeficiency syndrome. J. Infect. Dis..

[13] Keath E.J., Kobayashi G.S., Medoff G. (1992). Typing of *Histoplasma capsulatum* by restriction fragment length polymorphisms in a nuclear gene. J. Clin. Microbiol..

[14] Poonwan N., Imai T., Na M., Yazawa K., Mikami Y., Ando A., Nagata Y. (1998). Genetic analysis of *Histoplasma capsulatum* strains isolated from clinical specimens in Thailand by a PCR-based random amplified polymorphic DNA method. J. Clin. Microbiol..

[15] Reyes-Montes M.R., Bobadilla-Del Valle M., Martínez-Rivera M.A., Rodríguez-Arellanes G., Maravilla E., Sifuentes-Osornio J., Taylor M.L. (1999). Relatedness analyses of *Histoplasma capsulatum* isolates from Mexican patients with AIDS-associated histoplasmosis by using histoplasmin electrophoretic profiles and randomly amplified polymorphic DNA patterns. J. Clin. Microbiol..

[16] Muniz M. de M., Pizzini C.V., Peralta J.M., Reiss E., Zancopé-Oliveira R.M. (2001). Genetic diversity of *Histoplasma capsulatum* strains isolated from soil, animals, and clinical specimens in Rio de Janeiro State, Brazil, by a PCR-based random amplified polymorphic DNA assay. J. Clin. Microbiol..

[17] Kasuga T., Taylor J.W., White T.J. (1999). Phylogenetic relationships of varieties and geographical groups of the human pathogenic fungus *Histoplasma capsulatum* Darling. J. Clin. Microbiol..

[18] Jiang B., Bartlett M., Allen S.D., Smith J.W., Wheat L.J., Connolly P.A., Lee C.H. (2000). Typing of *Histoplasma capsulatum* isolates based on nucleotide sequence variation in the Internal Transcribed Spacer regions of rRNA genes. J. Clin. Microbiol..

[19] Carter D.A., Taylor J.W., Dechairo B., Burt A., Koenig G.L., White T.J. (2001). Amplified single-nucleotide polymorphisms and a (GA)_n_ microsatellite marker reveal genetic differentiation between populations of *Histoplasma capsulatum* from the Americas. Fungal Genet. Biol..

[20] Kasuga T., White T.J., Koenig G., McEwen J., Restrepo A., Castañeda E., Da Silva Lacaz C., Heins-Vaccari E.M., De Freitas R.S., Zancopé-Oliveira R.M. (2003). Phylogeography of the fungal pathogen *Histoplasma capsulatum*. Mol. Ecol..

[21] Taylor M.L., Chávez-Tapia C.B., Rojas-Martínez A., Reyes-Montes M.R., Bobadilla-Del Valle M., Zúñiga G. (2005). Geographical distribution of genetic polymorphism of the pathogen *Histoplasma capsulatum* isolated from infected bats, captured in a central zone of Mexico. FEMS Immunol. Med. Microbiol..

[22] De Muniz M.M., Morais P.M.S., Meyer W., Nosanchuk J.D., Zancopé-Oliveira R.M. (2010). Comparison of different DNA-based methods for molecular typing of *Histoplasma capsulatum*. Appl. Environ. Microbiol..

[23] Balajee S.A., Hurst S.F., Chang L.S., Miles M., Beeler W., Hale C., Kasuga T., Benedict K., Chiller T., Lindsley M.D. (2013). Multilocus sequence typing of *Histoplasma capsulatum* in formalin-fixed paraffin-embedded tissues from cats living in non-endemic regions reveals a new phylogenetic clade. Med. Mycol..

[24] Galo C., Sanchez A.L., Fontecha G.A. (2013). Genetic diversity of *Histoplasma capsulatum* isolates from Honduras. Sci. J. Microbiol..

[25] Vite-Garín T., Estrada-Bárcenas D.A., Cifuentes J., Taylor M.L. (2014). The importance of molecular analyses for understanding the genetic diversity of *Histoplasma capsulatum*: An overview. Rev. Iberoam. Micol..

[26] Teixeira M.M., Patané J.S.L., Taylor M.L., Gómez B.L., Theodoro R.C., de Hoog S., Engelthaler D.M., Zancopé-Oliveira R.M., Felipe M.S.S., Barker B.M. (2016). Worldwide phylogenetic distributions and population dynamics of the genus *Histoplasma*. PLoS Negl. Trop. Dis..

[27] Sepúlveda V.E., Márquez R., Turissini D.A., Goldman W.E., Matute D.R. (2017). Genome sequences reveal cryptic speciation in the human pathogen *Histoplasma capsulatum*. MBio.

[28] Maxwell C.S., Sepúlveda V.E., Turissini D.A., Goldman W.E., Matute D.R. (2018). Recent admixture between species of the fungal pathogen *Histoplasma*. Evol. Let..

[29] Taylor J.W., Jacobson D.J., Kroken S., Kasuga T., Geiser D.M., Hibbett D.S., Fisher M.C. (2000). Phylogenetic species recognition and species concepts in fungi. Fungal Genet. Biol..

[30] Tibayrenc M. (1996). Towards a unified evolutionary genetics of microorganisms. Annu. Rev. Microbiol..

[31] Rodríguez-Arellanes G., Pérez-Mejía A., Duarte-Escalante E., Taylor M.L. (1998). Organización de la colección de cepas de *Histoplasma capsulatum* del Laboratorio de Inmunología de Hongos, Facultad de Medicina, UNAM. Rev. Inst. Nal. Enf. Resp. Mex..

[32] Gannon W.L., Sikes R.S. (2007). The Animal Care and Use Committee of the American Society of Mammalogists: Guidelines of the American Society of Mammalogists for the use of wild mammals in research. J. Mammal..

[33] Altschul S.F., Gish W., Miller W., Myers E.W., Lipman D.J. (1990). Basic local alignment search tool. J. Mol. Biol..

[34] Farris J.S., Källersjö M., Kluge A.G., Bult C. (1994). Testing significance of incongruence. Cladistics.

[35] Goloboff P.A., Farris J.S., Nixon K.C. (2008). TNT, a free program for phylogenetic analysis. Cladistics.

[36] Nixon K.C. (1999). The parsimony ratchet, a new method for rapid parsimony analysis. Cladistics.

[37] Silvestro D., Michalak I. (2012). RaxmlGUI: A graphical front-end for RAxML. Org. Divers. Evol..

[38] Ronquist F., Teslenko M., van der Mark P., Ayres D.L., Darling A., Hohnä S., Larget B., Liu L., Suchard M.A., Huelsenbeck J.P. (2012). MrBayes 3.2: Efficient Bayesian phylogenetic inference and model selection across a large model space. Syst. Biol..

[39] Posada D. (2008). JModelTest: Phylogenetic model averaging. Mol. Biol. Evol..

[40] Heled J., Drummond A.J. (2010). Bayesian inference of species trees from multilocus data. Mol. Biol. Evol..

[41] Drummond A.J., Suchard M.A., Xie D., Rambaut A. (2012). Bayesian phylogenetics with BEAUti and the BEAST 1.7. Mol. Biol. Evol..

[42] Bandelt H.J., Forster P., Röhl A. (1999). Median-joining networks for inferring intraspecific phylogenies. Mol. Biol. Evol..

[43] Librado P., Rozas J. (2009). DnaSP v5: A software for comprehensive analysis of DNA polymorphism data. Bioinformatics.

[44] Mayden R.L., Oaridge M.F., Dawah H.A., Wilson M.R. (1997). A hierarchy of species concepts: The denouement in the saga of the species problem. Species. The Units of Biodiversity.

[45] Arita H.T., Ortega J., Ceballos G., Oliva G. (2005). Tadarida brasiliensis (I. Geoffroy, 1824). Los Mamíferos Silvestres de México.

[46] Iñiguez-Dávalos L.I., Ceballos G., Oliva G. (2005). *Mormoops megalophylla* (Peters, 1864). Los Mamíferos Silvestres de México.

[47] Russel A.L., Medellin R.A., McCracken G.F. (2005). Genetic variation and migration in the Mexican free-tailed bat *Tadarida brasiliensis mexicana*. Mol. Ecol..

[48] Hipp A.L., Hall J.C., Sytsma K.J. (2004). Congruence versus phylogenetic accuracy: Revisiting the incongruence length difference test. Syst. Biol..

[49] Sites J.W., Marshall J.C. (2004). Operational criteria for delimiting species. Annu. Rev. Ecol. Evol. Syst..

