# Supplementary figures and images for: Histoplasma capsulatum Isolated from Tadarida brasiliensis Bats Captured in Mexico Form a Sister Group to North American Class 2 Clade
Source: J Fungi (Basel). 2021 Jun 30;7(7):529. doi: 10.3390/jof7070529 (PMC8305335; doi:10.3390/jof7070529)

arf

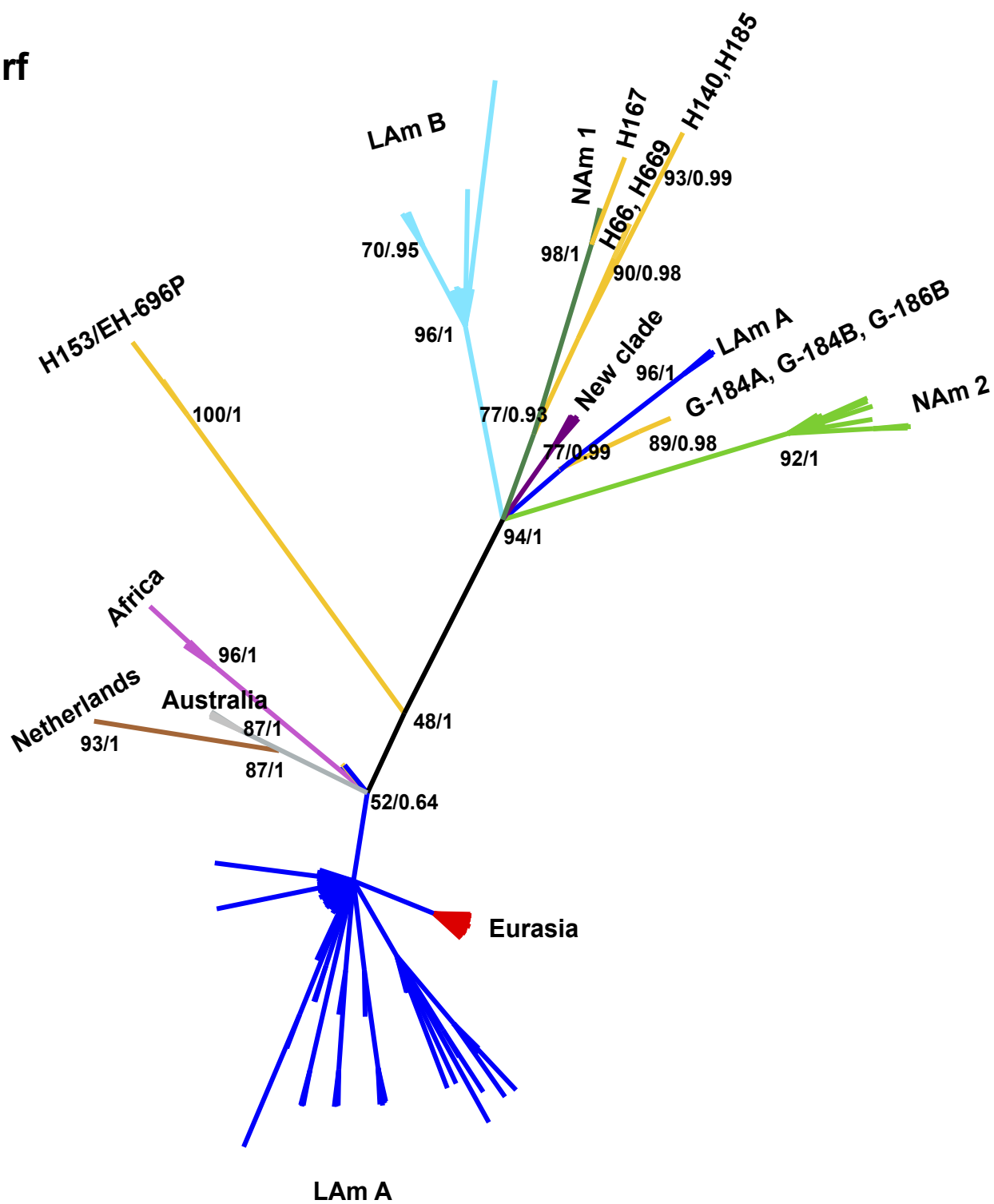

Supplement: Supplementary file 1 [file jof-07-00529-s001.zip › Figures Vite Garín et al/Figure S1 Vite-Garín et al. 10-04-21.pdf]

H-anti

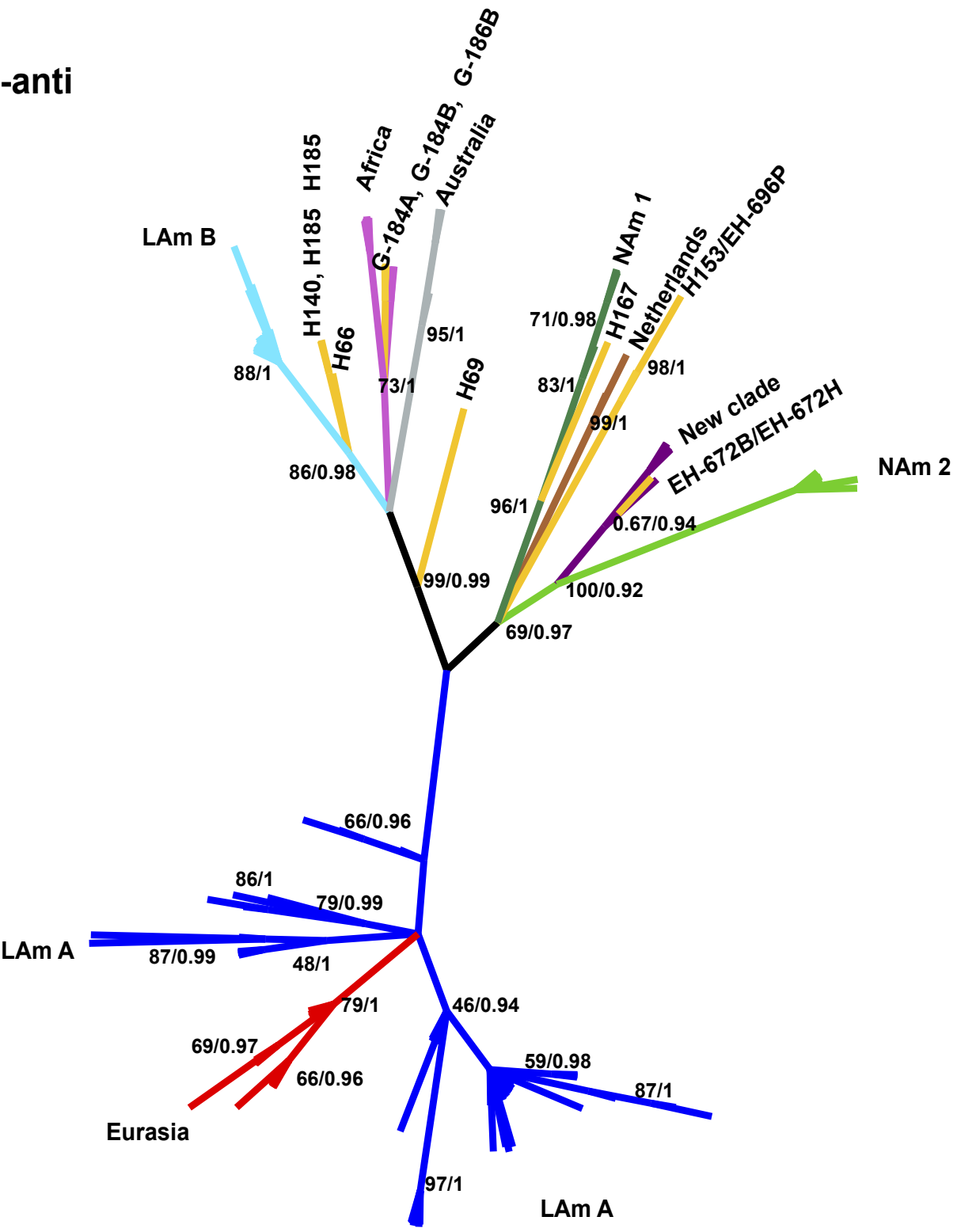

Supplement: Supplementary file 1 [file jof-07-00529-s001.zip › Figures Vite Garín et al/Figure S2 Vite-Garin et al. 10-04-21.pdf]

ole1

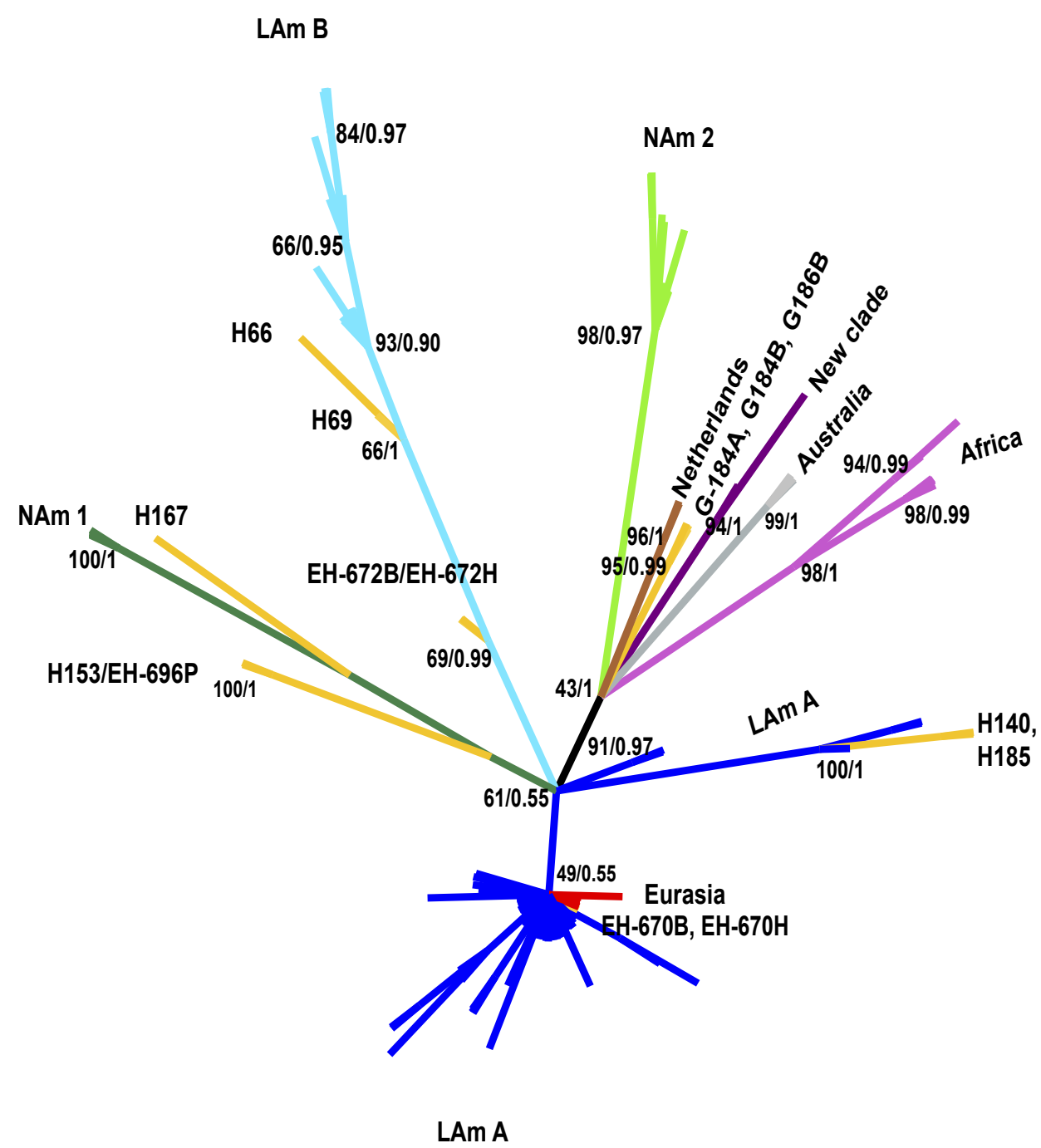

Supplement: Supplementary file 1 [file jof-07-00529-s001.zip › Figures Vite Garín et al/Figure S3 Vite-Garín et al. 10-04-21.pdf]

tub1

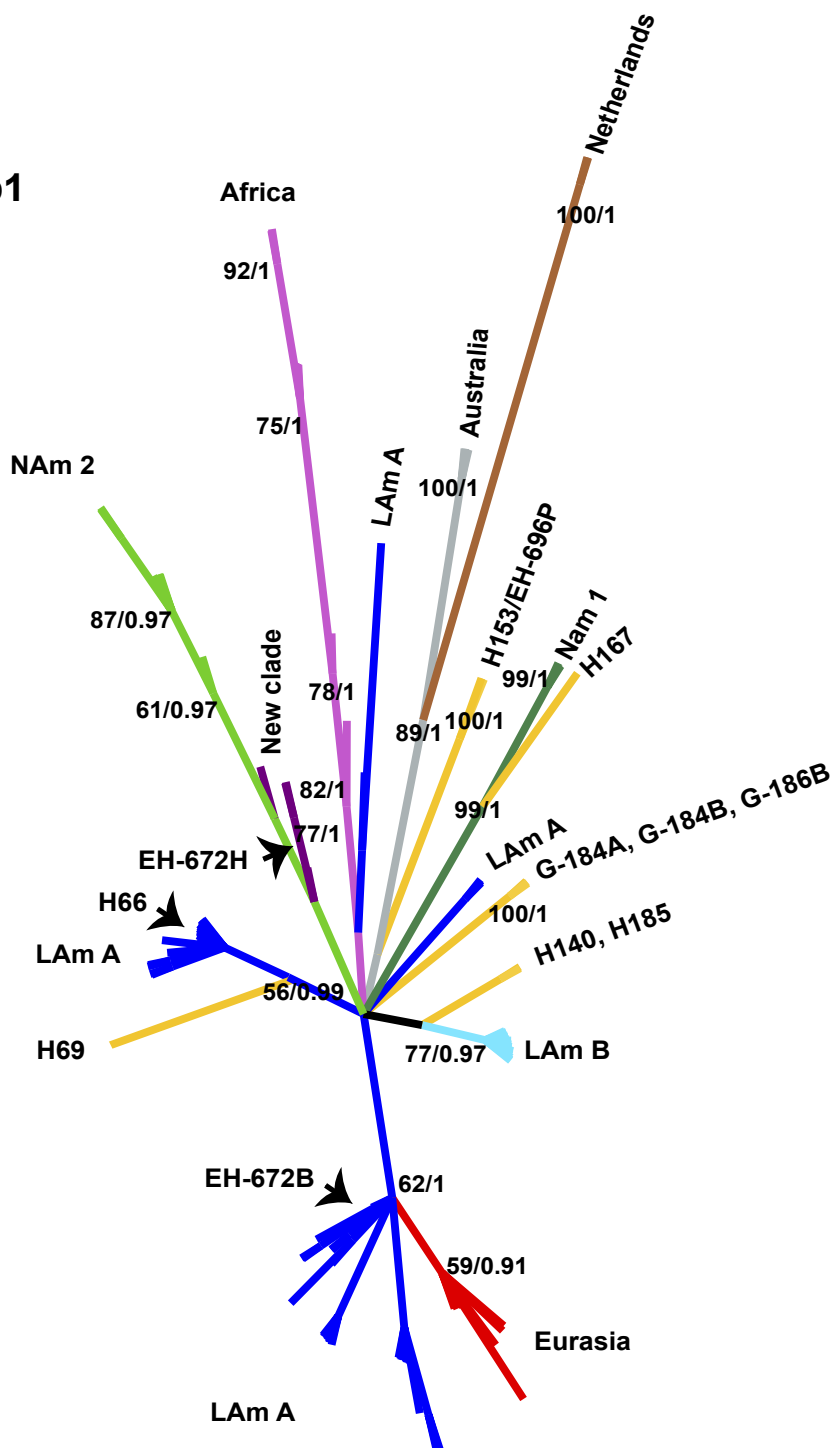

Supplement: Supplementary file 1 [file jof-07-00529-s001.zip › Figures Vite Garín et al/Figure S4 Vite-Garín et al. 10-04-21.pdf]

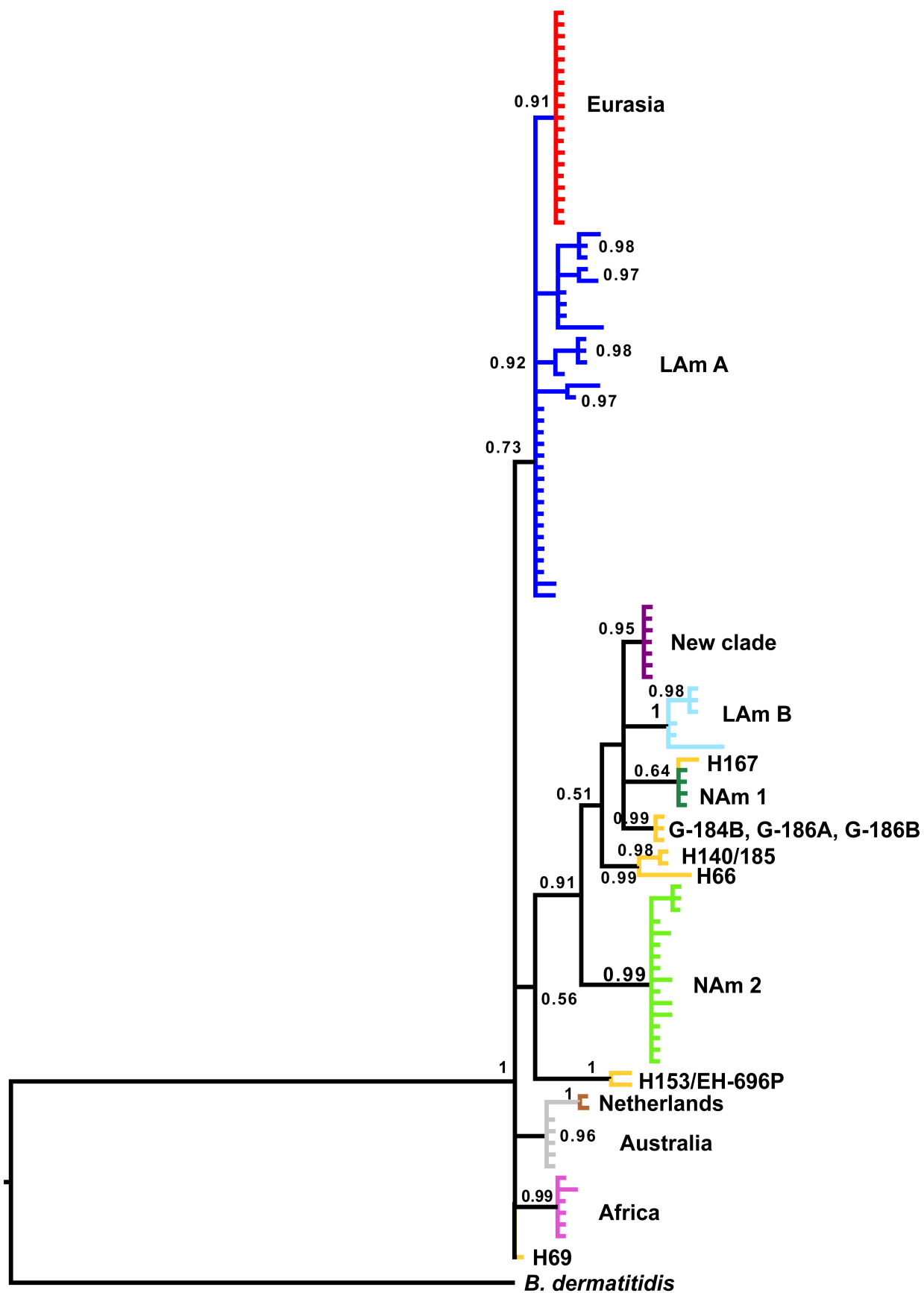

Supplement: Supplementary file 1 [file jof-07-00529-s001.zip › Figures Vite Garín et al/Figure S5 Vite-Garin et al. 10-04-21.pdf]

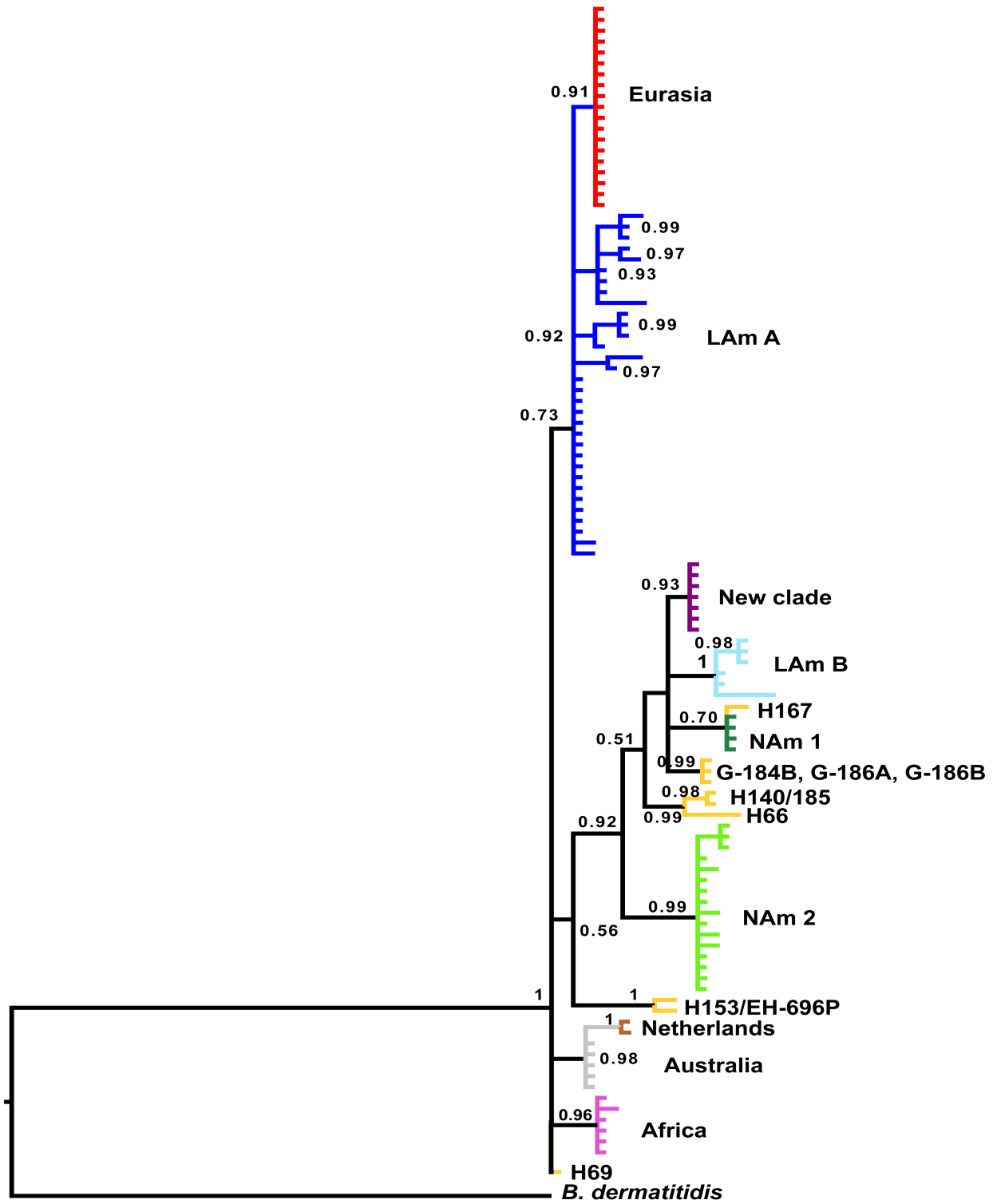

Supplement: Supplementary file 1 [file jof-07-00529-s001.zip › Figures Vite Garín et al/Figure S6 Vite-Garín et al. 10-04-21.pdf]

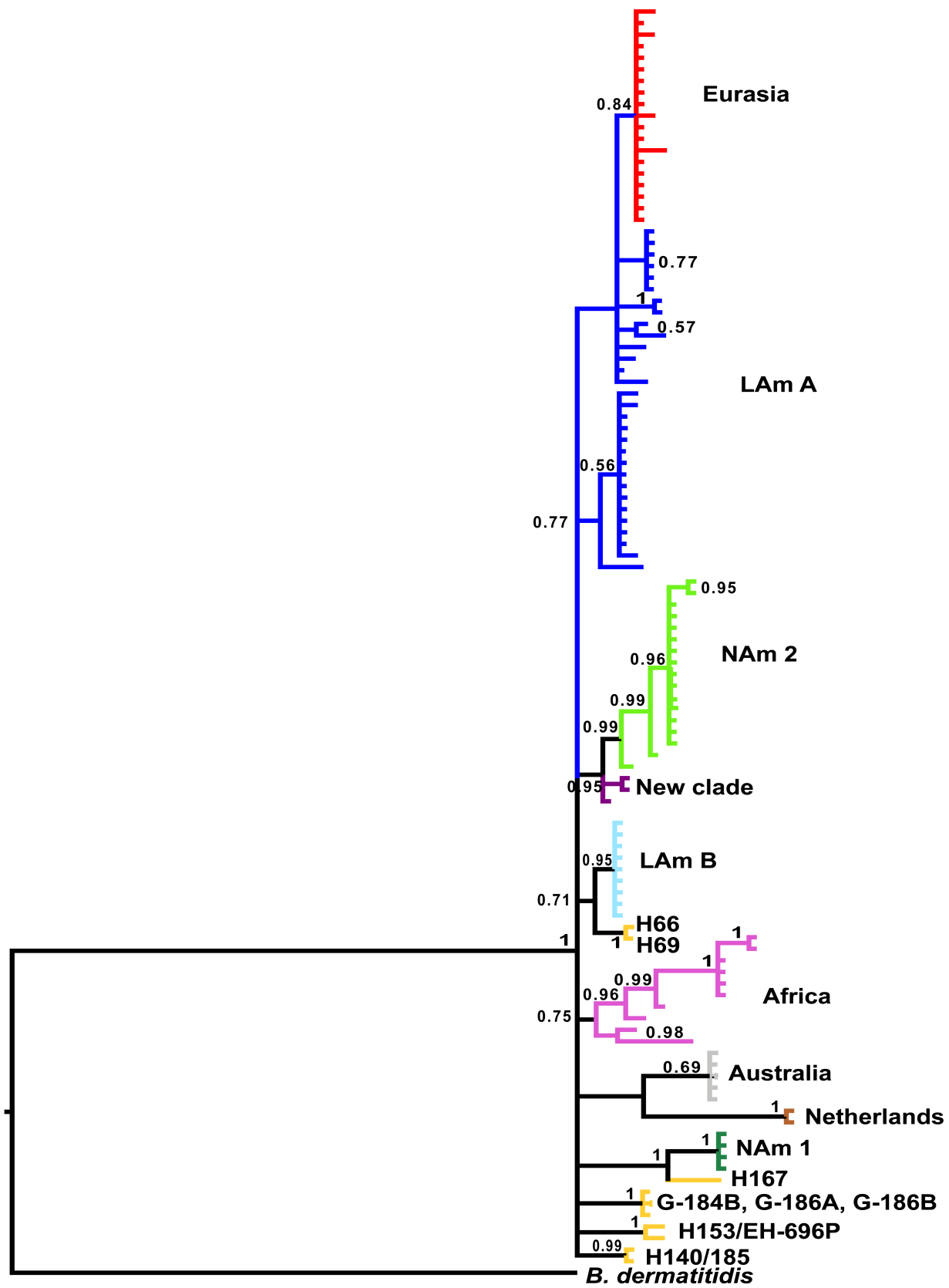

Supplement: Supplementary file 1 [file jof-07-00529-s001.zip › Figures Vite Garín et al/Figure S7 Vite-Garín et al. 10-04-21.pdf]

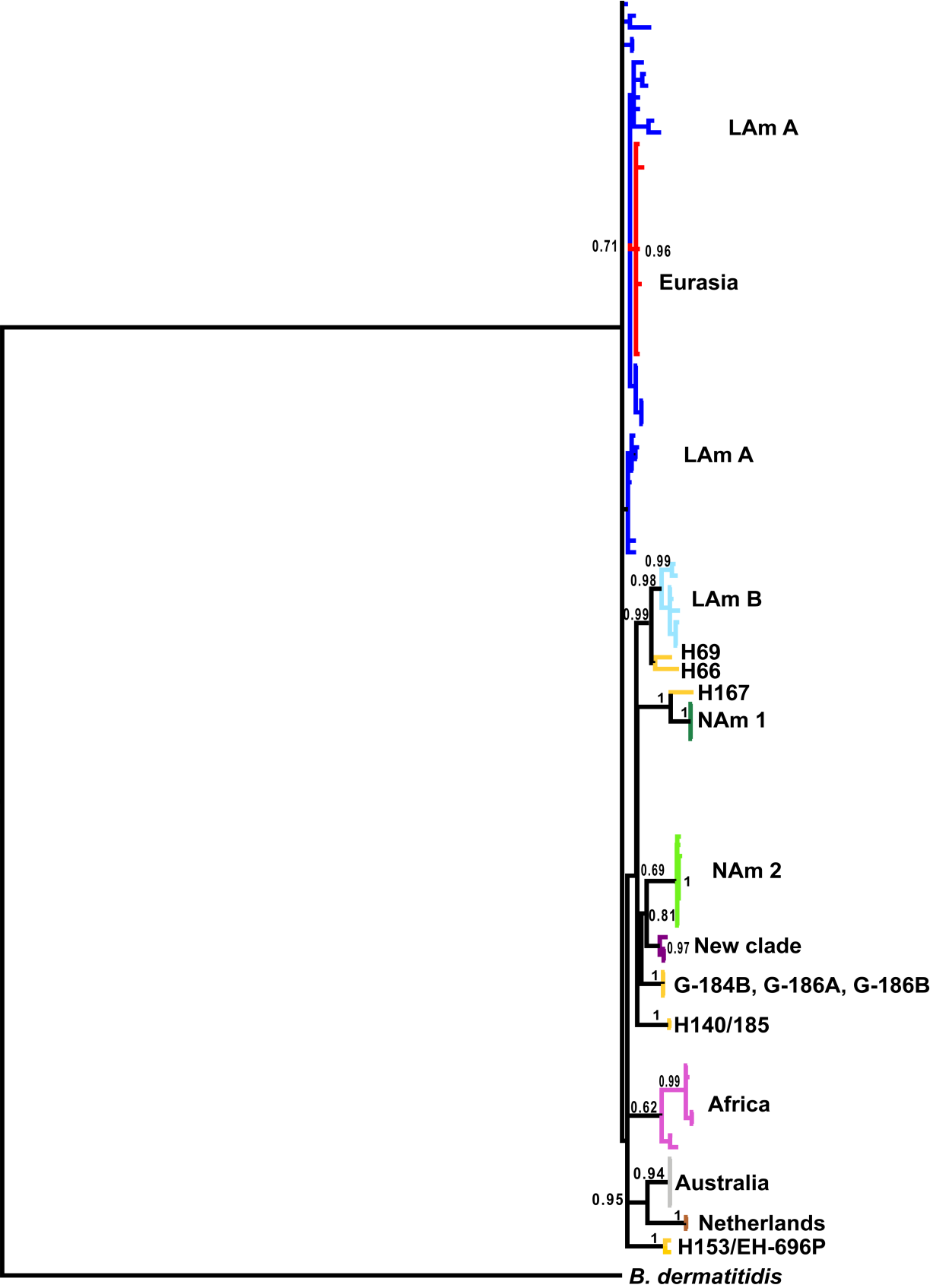

Supplement: Supplementary file 1 [file jof-07-00529-s001.zip › Figures Vite Garín et al/Figure S8 Vite-Garín et al. 10-04-21.pdf]
